# WNT5A transforms intestinal CD8αα^+^ IELs into an unconventional phenotype with pro-inflammatory features

**DOI:** 10.1186/s12876-015-0402-3

**Published:** 2015-12-10

**Authors:** Di Zhao, Antao Xu, Zhanghan Dai, Jiangchen Peng, Mingming Zhu, Jun Shen, Qing Zheng, Zhihua Ran

**Affiliations:** Division of Gastroenterology and Hepatology, Shanghai Institute of Digestive Disease, Ren Ji Hospital, School of Medicine, Shanghai Jiaotong University, 145 Middle Shandong Road, Shanghai, 200001 China

**Keywords:** Ulcerative colitis, Intestinal intraepithelial lymphocytes, WNT, Epithelial immunity

## Abstract

**Background:**

Intestinal intraepithelial lymphocytes that reside within the epithelium of the intestine form one of the main branches of the immune system. A majority of IELs express CD8α homodimer together with other molecules associated with immune regulation. Growing evidence points to the WNT signaling pathway as a pivotal piece in the immune balance and focuses on its direct regulation in intestinal epithelium. Therefore we decided to investigate its role in IELs’ immune status determination.

**Method:**

DSS colitis was induced in male C57BL mice. IELs were isolated from colon samples using mechanical dissociation followed by percoll gradient purification and Magnetic-activated cell sorting. Phenotype and cytokine production and condition with Wnts were analyzed by flow cytometry, real-time PCR or ELISA. Proliferation of lymphocytes were evaluated using CFSE dilution. Cell responses after WNT pathway interference were also evaluated.

**Results:**

Non-canonical WNT pathway elements represented by FZD5, WNT5A and NFATc1 were remarkably elevated in colitis IELs. The non-canonical WNT5A skewed them into a pro-inflammatory category as measured by inhibitory cell surface marker LAG3, LY49E, NKG2A and activated marker CD69 and FASL. Gaining of a pro-inflammatory marker was correlated with increased IFN-γ production but not TNF whilst decreased TGF-β and IL-10. Both interrupting WNT5A/PKC pathway and adding canonical WNT stimulants could curtail its immune-activating effect.

**Conclusion:**

Canonical and non-canonical WNT signals act in opposing manners concerning determining CD8αα^+^ IELs immune status. Targeting non-canonical WNT pathway may be promising in tackling inflammatory bowel disease.

## Background

Inflammatory bowel disease especially ulcerative colitis (UC) is related to the overly activation of colonic epithelial immunity [1, 2]. The intestinal epithelium is composed of a monolayer of cells that, among other functions, provides a physical barrier between the antigen-laden lumen of the intestine and the sterile environment beyond the basal layer. A large and diverse community of immune cells populates the epithelium. The most prevalent and most studied of these cells include the intraepithelial lymphocytes (IELs), which are predominantly T cells and can be divided into two major subsets of natural and induced IELs based on the mechanisms by which they become activated and on the cognate antigens that they recognize [3]. The primary role of natural IELs seems to be ensuring the integrity of the intestinal epithelium and maintaining local immune quiescence. The majority of them express the CD8α homodimers which instead of serving as a T cell co-receptor similar to CD8αβ heterodimers on conventional CD8^+^ T cells, have been postulated to be a repressor of TCR signaling [4]. In addition, their ability to express tolerogenic molecules such as LY49E and TGF-β further accentuates features of immune regulation [5, 6].

The WNT family of proteins regulates cell proliferation and differentiation in normal tissues and in cancer [7]. Growing evidence points out that the WNT signaling pathway also acts as a pivotal piece in the immune balance thus increasing focuses have been set on its direct regulation in intestinal epithelium [8–11]. Upon activation by canonical WNTs, β-catenin translocates to the nucleus, where it associates with TCF/LEF family transcription factors to activate target gene expression. While WNT/β-catenin has been ascribed a tumor stimulating effect, it also seems to mediate anti-inflammatory effects [12]. At the same time studies based on the modulation of non-canonical WNT signaling downstream molecules suggest a potential pro-inflammatory role. Its induced transcription factor nuclear factor κB (NF-κB) is crucial for inflammation, immunity and tissue homeostasis [13], and appears to be a critical link between inflammation and cancer [14]. Interestingly, mounting evidence has shown that WNT/β-catenin can negatively regulate the expression of a group of pro-inflammatory target genes of NF-κB [15]. The high level of WNTs activity in colon and the ability of WNTs to help determine the immune status of effector cells prompt us to investigate the consequence they endow with IELs.

In this study, we assessed the direct effects of WNTs on the biology and functional responses of CD8αα^+^ IELs. We reported that WNT3A directly induced immune-regulatory cytokine production whereas WNT5A gave rise to pro-inflammatory cytokines in these cells. The canonical and non-canonical WNT ligands seemed to act in opposing manners when ablate canonical WNT co-receptor further enhanced WNT5A’s immune activating outcomes. Inhibiting WNT5A downstream effector can reverse pro-inflammatory phenotype of CD8αα^+^ IELs. Moreover, WNT5A appeared to induce a Th1-like effect in those IELs. The results suggest an important role for the WNT family in governing CD8αα^+^ IELs’ immune responses.

## Methods

### Mice model

4-week-old C57BL male mice were obtained from the Shanghai SLAC Laboratory Animal CO. LTD (Shanghai Laboratory Animal Center, Chinese Academy Sciences). They were housed in environmentally-controlled conditions (temperature 22 °C, with light cycle from 06:00 am to 6:00 pm). DSS colitis model were established as described elsewhere [16]. All animal experimentations were approved by the Institutional Animal Use and Care Committee of Shanghai University of Traditional Chinese Medicine.

### Cell purification

Intraepithelial lymphocytes were retained by mechanical dissociation from 10 weeks old wild-type C57BL or DSS induced colitis mice as described before [17]. In short, mice intestine tissue was dissected and cut into 5 mm pieces. Every gram of tissue was added into 5 ml of 5 mM EDTA/5 mM DTT solution to remove the mucous and epithelium. Solution was gently shaken at 150 rpm under 37 °C for 15 min. A total of 3 times of removals were carried out with dissociated cells collected after each conduct. Density gradient step was done by re-suspending cells in 40 % percoll and carefully laid on 80 % percoll solution and centrifuged at 500 rcf under 4 °C for 20 min. The viability of yielding cells was around 90 %. CD8αα^+^ lymphocytes were selected by magnetic activated cell sorting using Miltenyi products (130-094-973 and 130-104-075) as the manufacturer instructed. The purity of the CD8αα^+^ population was 85–95 %. Isolated cells were maintained in RPMI-1640 (life Techenologies) supplemented with fetal bovine serum (10 % v/v) (life Techenologies), 100 U/mL penicillin-streptomycin (life Techenologies), 2 mmol/L L-glutamine (life Techenologies), anti-CD3 (10 ug/ml) (BD Pharmingen) and anti-CD28 (2 ug/ml) (BD Pharmingen). JURKAT and EL4 cells were bought from ATCC and were cultured in RPMI 1640 supplement with the fetal bovine serum (10 % v/v).

### Abs, chemicals and ELISA reagents

The following Abs were used to detect human(h) or mouse(m) antigen for flowcytometry analysis: CD8α(m), Clone 53–6.7, BD Pharmingen; LAG3(m), Clone C9B7W, BD Pharmingen; LAG3(h), Clone 3DS223H, eBioscience; LY49E(m), Clone CM4, eBioscience; NKG2A(m), Clone 20d5, BD Pharmingen; NKG2A(h), Clone 131411, R&D Systems; FASL(m), Clone MFL3, eBioscience; FASL(h), Clone NOK-1, eBioscience; CD69(m), Clone H1.2 F3, BD Pharmingen; CD69(h), Clone L78, BD Pharmingen; Granzyme B(m), Clone NGZB eBioscience; Granzyme B(h), Clone GB11, BD Pharmingen; IL-4(m), Clone 11B11, BD Pharmingen; NKG2D(m), Clone CX5, BD Pharmingen; T-bet(m), Clone 04–46, BD Pharmingen; GATA3(m), Clone L50-823, BD Pharmingen.

Primary Abs used for Western blot analysis were: Anti-LRP6 antibody-N-terminal, Abcam; PKC-alpha [pT638] Rabbit Polyclonal Antibody, Life Technologies; Phospho-PKCα/β II (Thr638/641) Antibody, Cell Signaling; PKCdelta Antibody, Thermo Scientific Pierce Antibodies; Phospho-PKCdelta (Tyr311) Antibody, Cell Signaling; NFAT2 (D15F1) Rabbit mAb, Cell Signaling Technology; Anti-Human/Mouse GAPDH Purified, eBioscience.

Chemicals used in function analysis: Wnt3a (315–20, PeproTech); Wnt5a (645-WN-010, R&D Systems); BisindolylmaleimideI (G2911, Sigma-Aldrich); Cyclosporin A (30024, Sigma-Aldrich).

The following reagents were used for ELISA: Human IL-10 Quantikine ELISA Kit, Mouse IL-10 Quantikine ELISA Kit, Human TGF-beta 1 Quantikine ELISA Kit, Mouse/Rat/Porcine/Canine TGF-beta 1 Quantikine ELISA Kit, Human IFN-gamma Quantikine ELISA Kit, Mouse IFN-gamma Quantikine ELISA Kit, Human TNF-alpha Quantikine ELISA Kit and Mouse TNF-alpha Quantikine ELISA Kit. All were purchased from RnD systems.

### CFSE labeling and flowcytometry detection

T cells were labeled with CFSE (21888, Sigma-Aldrich) for 10 min at 37 °C, followed by 10 % FBS deactivation and extensive washing, prior to culture. Proliferation was assessed on D4 of culture. As for cytokine detection, on the last day of culture, cells were stimulated for 4–6 h with PMA (10 ng/ml) and ionomycin (1 mg/ml; both from peprotech) to enhance cytokine expression. Intracellular stainings were performed as the manufacturer indicated (554714, BD Pharmingen). Flowcytometry analysis was performed on an LSR II flow cytometer (BD Pharmingen).

### RNAi

A siRNA sequence targeting the mouse (sc-37234, Santa Cruz) or human (sc-37233, Santa Cruz) LRP6 were introduced into EL4 or JURKAT cells using Lipofectamine® RNAiMAX Reagent (Life Technologies) as indicated by the manufacturer’s instruction. The efficacy of knockdown was evaluated by rtPCR and western-blot.

### Real-time PCR analysis

Total cellular RNA from cells of interest was extracted using TRIzol (Life Technologies). We reverse transcribed the total amount of RNA and quantify the cDNA content as the kit manufacturer instructed (RR037, RR820, both from Takara). RNA samples were treated with RNase-free DNase I (Invitrogen) before reverse transcribed to eliminate contaminating genomic DNA. We determined the expression of the mRNAs for human (h) and/or mice (m) of interest using the primers listed in supplement Table 1. Expression was normalized to the level of β-actin in each sample.

**Table 1:** Primers used to determine the expression of the mRNAs for human (h) and/or mice (m) of interest

FZD1(h),	5’-TTGTGGGCATCACATCGGGCTT-3’,	5’-TGCAAAAGTCAGTGGCTGGCGA-3’;
FZD1(m),	5’-TTGTGGGCATCACATCGGGCTT-3’,	5’-TGCAAAAGTCAGTGGCTGGCGA-3’;
FZD2(h),	5’-AGTTCGGTTTTCAGTGGCCCGA-3’,	5’-TCACGCTCGCCCAGAAACTTGT-3’;
FZD2(m),	5’-ACGCGCAGAGAATGGGCAACAA-3’,	5’-AACGGAAGCGCCAAAGATCCCA-3’;
FZD3(h),	5’-TGTTCTCGGGATTTCCGGCCTT-3’,	5’-GGCTCATCACAATCTGGGAACCTAC-3’;
FZD3(m),	5’-TGCAGCTTTAGCAATGGAGCCCT-3’,	5’-ATCTTCAGGCCACGGGACACCAAA-3’;
FZD4(h),	5’-TGCAGGGTGGGATTTGAGCTGTGA-3’,	5’-AAACAGCAGACAGCGCACCACA-3’;
FZD4(m),	5’-TGCAAACTGGGGGTGTCTGCTA-3’,	5’-TGGTCACGTTGTAGCCGAGGTT-3’;
FZD5(h),	5’-AACAGCATCCGTGACCTGCACT-3’,	5’-TGCAGAGGGAGCAGGCAATTCT-3’;
FZD5(m),	5’-ATCCTCCGAGAGTTCTGTCCTTGG-3’,	5’-TCGTCTCCTTCTTCCCTTTGCCT-3’;
FZD7(h),	5’-CGTGCCAACGGCCTGATGTACTTT-3’,	5’-TGAAGTAGCAGCCCGACAGGAAGA-3’;
FZD7(m),	5’-TGCAGTCATGGCGTCGCTTCTA-3’,	5’-TTCGGCCACTGCCTTTGCCTTT-3’;
FZD9(h),	5’-ACCTGGTGCTGGGCAGTAGTTT-3’,	5’-GCCAGAAGTCCATGTTGAGGCGTT-3’;
FZD9(m),	5’-TGTGTGGTCCGCGTTGTGTT-3’,	5’-TGCAGCCTGTGTTTTCCAGACC-3’;
WNT1(h),	5’-TCGCGGGCAACAACCAAAGT-3’,	5’-ACGTTCACAATACCCCACCATCGG-3’;
WNT1(m),	5’-AACCGCAGCACAGAACCAGCAA-3’,	5’-AACCGCAGCACAGAACCAGCAA-3’;
WNT2(h),	5’-AAGATGGGAAGCGCCAAGGACA-3’,	5’-TTACAGCCTTCCTGCCAGCTCT-3’;
WNT2(m),	5’-AGGTCAGCTCTTCATGGTGGTACA-3’,	5’-AGGGTGTTGCAGTTCCAGCGAT-3’;
WNT3A(h),	5’-TGTTGGGCCACAGTATTCCTCCCT-3’,	5’-AAAGGCCGACTCCCTGGTAGCTTT-3’;
WNT3A(m),	5’-AGCCTGCTGTTGAGGCAATGGT-3’,	5’-AGCGCGAACGCAAAGTTCCA-3’;
WNT5A(h),	5’-ATTCTGGCTCCACTTGTTGCTCGG-3’,	5’-CCTAGCGACCACCAAGAATTGGCTT-3’;
WNT5A(m),	5’-ACGCCTGTGCAACAAGACCTCA-3’,	5’-AAGGGCAGGCACACCACTATTTGC-3’;
WNT11(h),	5’-AGGATGTGGCTGCTGACCTCAA-3’,	5’-TGTTGCACTGCCTGTCTTGTGTCC-3’;
WNT11(m),	5’-TGTTGCACTGCCTGTCTTGTGTCC-3’,	5’-TGGAGCGGAATCCTGTGTTCCCAA-3’;
DKK1(h),	5’-AACTCGGTTCTCAATTCCAACGCT-3’,	5’-GGTACGGCTGGTAGTTGTCAATGG-3’;
DKK1(m),	5’-TGCATGAGGCACGCTATGTGCT-3’,	5’-ACGGAGCCTTCTTGTCCTTTGGT-3’;
LRP6(h),	5’-TGGATCAACCCAGAGCTATTGCCTT-3’,	5’-ACCACTGCCTGCCGATTTGTT-3’;
LRP6(m),	5’-TGGATCAACCCAGAGCTATTGCCTT-3’,	5’-ACTGCCTGCCGGTTTGTTCCAT-3’;
APC(h),	5’-ACTCGGAAATGGGGTCCAAGGGTA-3’,	5’-TGACCGCAGTTTTACTCCAGGGAA-3’;
APC(m),	5’-TGCACAGCGAAGAATAGCCAGGA-3’,	5’-TGCGGTGTTGCTTTCTGCCACT-3’;
TCF1(h),	5’-ATGTGACCCAGAGCCCCTTCAT-3’,	5’-TCTGAGGTGAAGACCTGCTTGGTG-3’;
TCF1(m),	5’-AGCCGTGGTGGAGTCACTTCTT-3’,	5’-TGGGTGAATTGCTGAGCCACCT-3’;
LEF1(h),	5’-CAGATTCTTGGCAGAAGGTGGCAT-3’,	5’-AGCTGTCATTCTTGGACCTGTACCT-3’;
LEF1(m),	5’-CTGCCTACATCTGAAACATGGTGGT-3’,	5’-TGCTGTCAGTGTTCCTTGGGGT-3’;
GSK3B(h),	5’-ATTTCACCTCAGGAGTGCGGGTCT-3’,	5’-TGCAGGTGTGTCTCGCCCATTT-3’;
GSK3B(m),	5’-ACCAAATGGGCGAGACACACCT-3’,	5’-TTAGTATCTGAGGCTGCTGTGGCGT-3’;
CTNNB1(h),	5’-TCGAAATCTTGCCCTTTGTCCCGC-3’,	5’-ACCCCCTCCACAAATTGCTGCT-3’;
CTNNB1(m),	5’-TCCTTGCTCGGGACGTTCACAA-3’,	5’-TGCGTATGTTGCCACGCCTTCA-3’;
DVL1(h),	5’-ATCTCGCCACCCTGAACCTCAACA-3’,	5’-TGCTTTTGCTCCCTTCACTCTGCTG-3’;
DVL1(m),	5’-TGCTCTTGCTCCCTTCACTCTGCT-3’,	5’-TGTCTTTGGCGACCTGTGCAGT-3’;
NFATC1(h),	5’-TCAGCGGAGGAAGAACACTATGGC-3’,	5’-ACAGGCCCAAGCACGAGGTTAT-3’;
NFATC1(m),	5’-TCGTGGAGAAGCAGAGCACAGACA-3’,	5’-CCACCAGGGAATTTGGCTTGCACA-3’;
IL-10(h),	5’-TCCTTGCTGGAGGACTTTAAGGGTT-3’,	5’-AAGGCATTCTTCACCTGCTCCACG-3’;
IL-10(m),	5’-TGCACTACCAAAGCCACAAGGCA-3’,	5’-CGACTGGGAAGTGGGTGCAGTTAT-3’;
TGF-β(h),	5’-ATGATCGTGCGCTCCTGCAA-3’,	5’-TGACACAGAGATCCGCAGTCCT-3’;
TGF-β(m),	5’-TCGACATGGAGCTGGTGAAACGGA-3’,	5’-TAGATGGCGTTGTTGCGGTCCA-3’;
IFN-γ(h),	5’-ACAAGGCTTTATCTCAGGGGCCA-3’,	5’-GCACTGGCTCAGATTGCAGGCATA-3';
IFN-γ(m),	5’-ACGCTACACACTGCATCTTGGCT-3’,	5’-CACCATCCTTTTGCCAGTTCCTCC-3’;
TNF(h),	5’-AGCCCATGTTGTAGCAAACCCTC-3’,	5’-ATGGTGTGGGTGAGGAGCACAT-3’;
TNF(m),	5’-AGCACAGAAAGCATGATCCGCGA-3’,	5’-TGGTGGTTTGTGAGTGTGAGGGT-3’;
IL-2(m),	5’-TTGTCAACAGCGCACCCACTTC-3’,	5’-TTCAATTCTGTGGCCTGCTTGGGC-3’;
IL-15(m),	5’-TCTGCGCCCAAAAGACTTGCAG-3’,	5’-TCAAGGTGGATTCTCTCTGAGCTGT-3’;
IL-2RB(m),	5’-ATGTCACAACCTGCCACGTCCA-3’,	5’-AGGCGAAGGTTGTCAAAGGGATGG-3’;
IL-15RA(m),	5’-GGCCTGGTACATCAAATCAAGGCAG-3’,	5’-ACATTGGGCTTTCTCCTGTGTCCA-3’;
IL-12(m),	5’-TCCCGAAACCTGCTGAAGACCA-3’,	5’-CCAGGCAACTCTCGTTCTTGTGT-3’;
IL-4(m),	5’-ACGTCCTCACAGCAACGAAGAACA-3’,	5’-TCCAGGCATCGAAAAGCCCGAA-3’;
IL-22(m),	5’-AGCCATACATCGTCAACCGCAC-3’,	5’-TGAGTTTGGTCAGGAAAGGCACC-3’;
C-MYC(m),	5’-AGCAGCGACTCTGAAGAAGAGCA-3’,	5’-AGCACTTGCGGTTGTTGCTGAT-3’;
PKCα(m),	5’-TGGGAAGGTGATGCTTGCTGAC-3’,	5’-AGCTGTGTCAGAAATGGCGGCT-3;
β-actin(h),	5’-TGGCATCCACGAAACTACCTTCAAC-3’,	5’-TCTTGATCTTCATTGTGCTGGGTGC-3’;
β-actin(m),	5’-AGCTGAGAGGGAAATCGTGCGT-3’,	5’-TCCATACCCAAGAAGGAAGGCTGGA-3’

### Western blot

We lysed cells in RIPA buffer (Beyotime) supplemented with protease and phosphatase inhibitors (Beyotime). Equal amounts of protein was loaded on SDS PAGE and blotted on nitrocellulose (Millipore). Membranes were blocked and probed with Abs for LRP6 (1:1000), PKCα (1:2000), Phospho-PKCα/β (1:1000), PKCδ (1:2000), Phospho-PKCδ (1:1000), NFATc1 (1:2000) and GAPDH (1:2000). Finally, proteins were visualized using ECL system (Pierce).

### Statistical analysis

Data is presented as mean values ± SEM, unless otherwise indicated. Statistical significance between sets of data was assessed with the two-tailed unpaired Student *t* test.

### Ethical approval and consent

All animal experimentations were approved by the Institutional Animal Use and Care Committee of Shanghai University of Traditional Chinese Medicine.

## Results

### Expression of WNT regulated genes were influenced by colitis status in intraepithelial cells

**Fig. 1:**
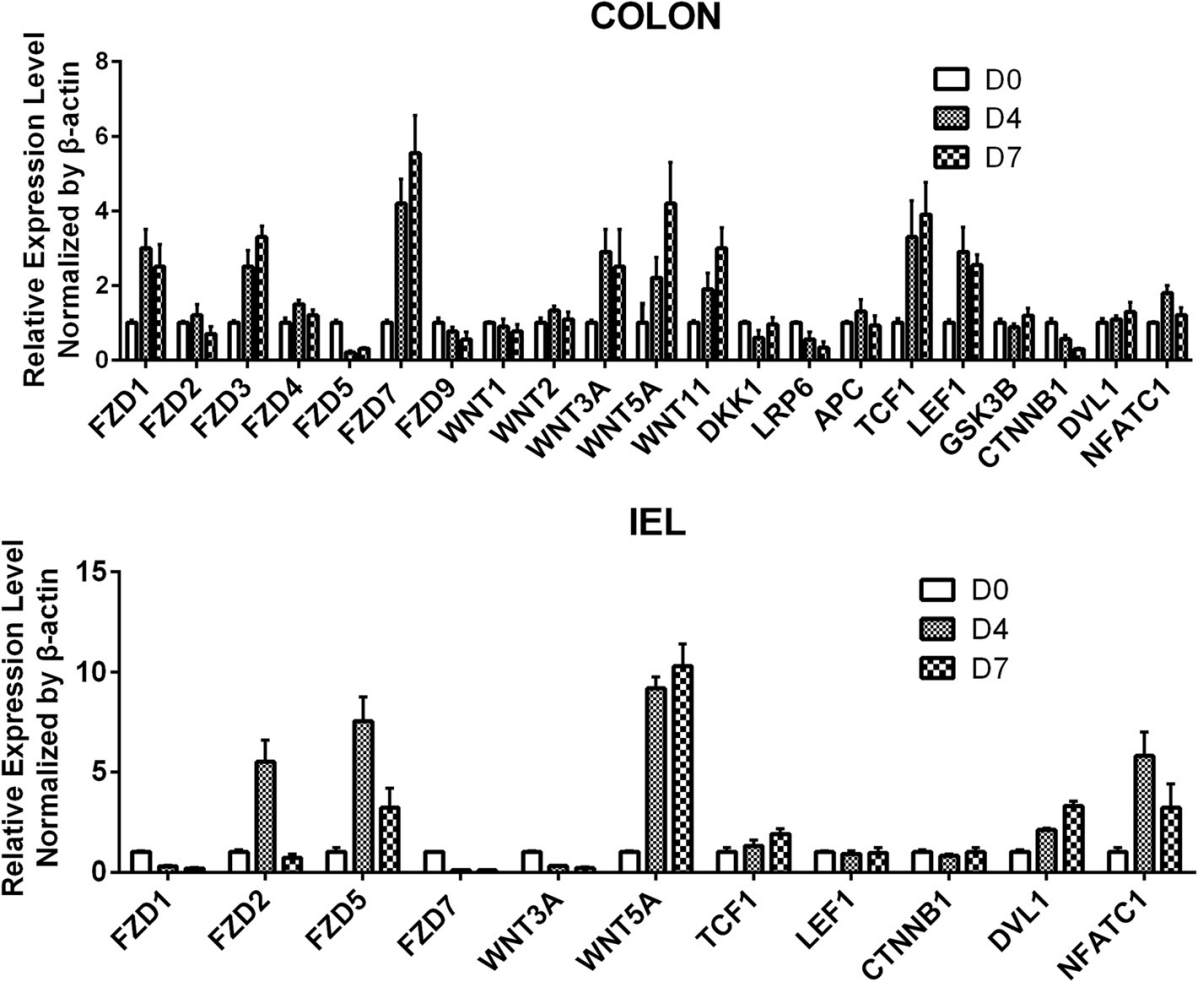
RT-PCR analysis of WNT pathway genes of colon tissues (up) and isolated IELs (down). Cells were isolated from DSS mice models and MACS-enriched. IELs represent total CD3ε + cells. mRNA expression was compared to the expression observed in healthy counterparts. Real-time PCR was performed in duplicate and samples were normalized to β-actin expression, with standard deviations indicated. D4: the fourth day of initiating DSS treatment, D7: the seventh day of initiating DSS treatment

To analyze the WNT signaling pathway elements’ expression levels in IELs during DSS colitis, we used rtPCR to profile the expression of WNT related genes in both distal colon tissue and isolated large intestine IELs. Albeit no statistically significant change was observed in the en-bloc resection, expression of several WNT canonical and non-canonical signaling elements were affected in IELs from inflamed colon as compared to healthy controls. Canonical WNT signaling pathway components FZD1, FZD7 and WNT3A dropped in response to the DSS treatment from day 4 to day 7, non-canonical WNT ligand WNT5A, receptor FZD2 and FZD5 and downstream NFATc1, quite on the contrary, were upregulated in IELs (Fig. 1). The transcription factor NFATc1 was reported to play an essential role in the pathogenesis of inflammatory bowel disease and augment a number of inflammation-associated genes. All those mRNA expression modulations suggest an involvement of WNT signaling pathway in IELs’ immuno-activation.

### The Canonical and non-canonical WNT ligands act in divergent ways in regulating CD8αα^+^ IELs immune response

**Fig. 2:**
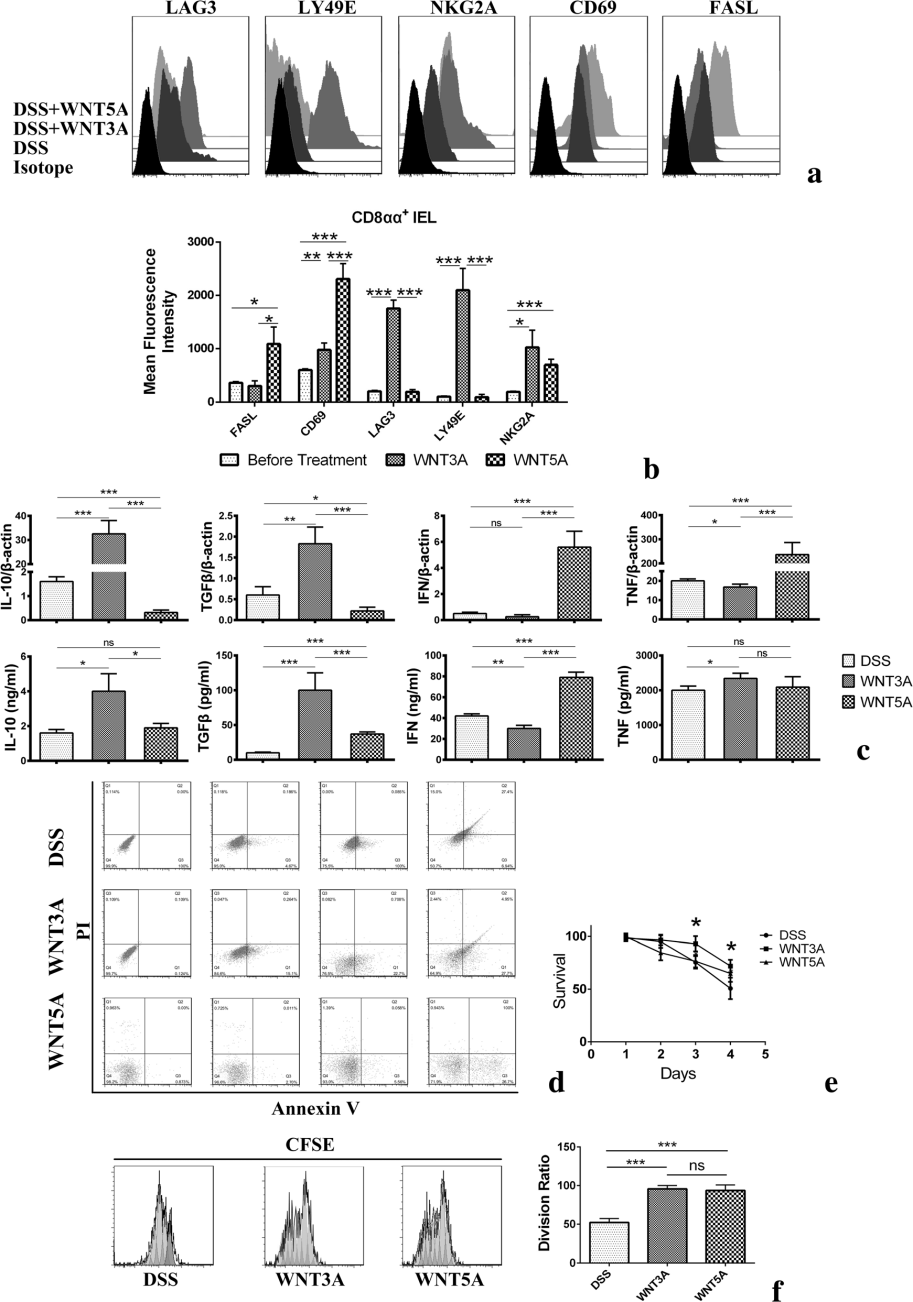
Markers and cytokines expression by CD8αα^+^. Sorted intestinal CD8αα^+^ IELs from DSS colitis mice were treated with 0.4 ng/ml WNT3A or 0.4 μg/ml WNT5A for 24 h as indicated. **a** Expression of surface markers in CD8αα^+^ cells. **b** Mean fluorescence intensity (MFI) was expressed as means ± SEM. Data represents three mice from two individual experiments. **c** Expression of cytokines in terms of mRNA (up) and protein quantity in supernatants (down). Conditioned with WNTs reduces apoptosis and enhances proliferation of CD8αα^+^ IELs. **d** Survival assessed by annexin V/PI staining for CD8αα^+^ IELs. **e** Statistical analysis for annexin V/PI-negative cells following treatment with results of three repeat experiments. **f** CFSE-labeled CD8αα^+^ IELs cultured for 4 days conditioned with WNTs. Data presented with means ± SEM from three independent experiments are shown. *p < 0.05, **p < 0.01, ***p < 0.005,ns: None-significant

We next performed a phenotypic analysis of CD8αα^+^ cells within the IELs gate after canonical or non-canonical WNT stimulants. CD8αα^+^ cells were found in the epithelium of the colon and constituted nearly 50 % of all CD45^+^CD3ε^+^ cells present within the epithelium. Isolated CD8αα^+^ IELs were cultured in medium alone or in the presence of WNT3A or WNT5A. Culturing with canonical WNT stimulants rapidly upregulated immune-regulating surface marker LAG3, LY49E and NKG2A while decreased activating marker FASL and CD69. At the same time, non-canonical ligand had noticeably augmented FASL and CD69, while had no effect on LAG3 and LY49E and only moderately upregulated NKG2A. Thus, the WNT signals acted on the CD8αα^+^ IELs on a dichotomous way when canonical signals seemed to allow those cells retain a tolerogenic phenotype (Fig. 2a and b).

Further analysis was performed on the ability of WNT3A and WNT5A to induce cytokine production in freshly isolated CD8αα^+^ IELs from mouse colon. Overnight stimulation of IELs with recombinant WNT3A stimulated secretion of the immune regulatory cytokine IL-10 and TGF-β. WNT5A also induced TGF-β yet to a lesser degree. The inflammatory mediator IFN can only be induced by WNT5A. Interestingly, though increased on mRNA level by WNT5A, tumor necrosis factor (TNF) protein expression was not modulated by either WNT5A or WNT3A. Thus, WNTs can promote CD8αα^+^ IELs production of immune regulatory cytokines (Fig. 2c).

Because WNT signals regulate the survival of lymphoid cells, we next examined whether WNTs were involved in CD8αα^+^ IELs survival. The viability of mechanically dissociated IELs dropped quickly during ex vivo culture, on the fourth day, the overall survival dropped to around 50 %. As shown in Fig. 2d and e, WNT5A conditioned cells were remarkably rigorous as determined by annexin V/PI staining. On the fourth day, around 70 % of IELs were still viable. WNT3A can also moderately enhance ex vivo cell viability. Both WNT3A and WNT5A could augment IELs proliferation as assessed by CFSE dilution assay (Fig. 2f).

**Fig. 3:**
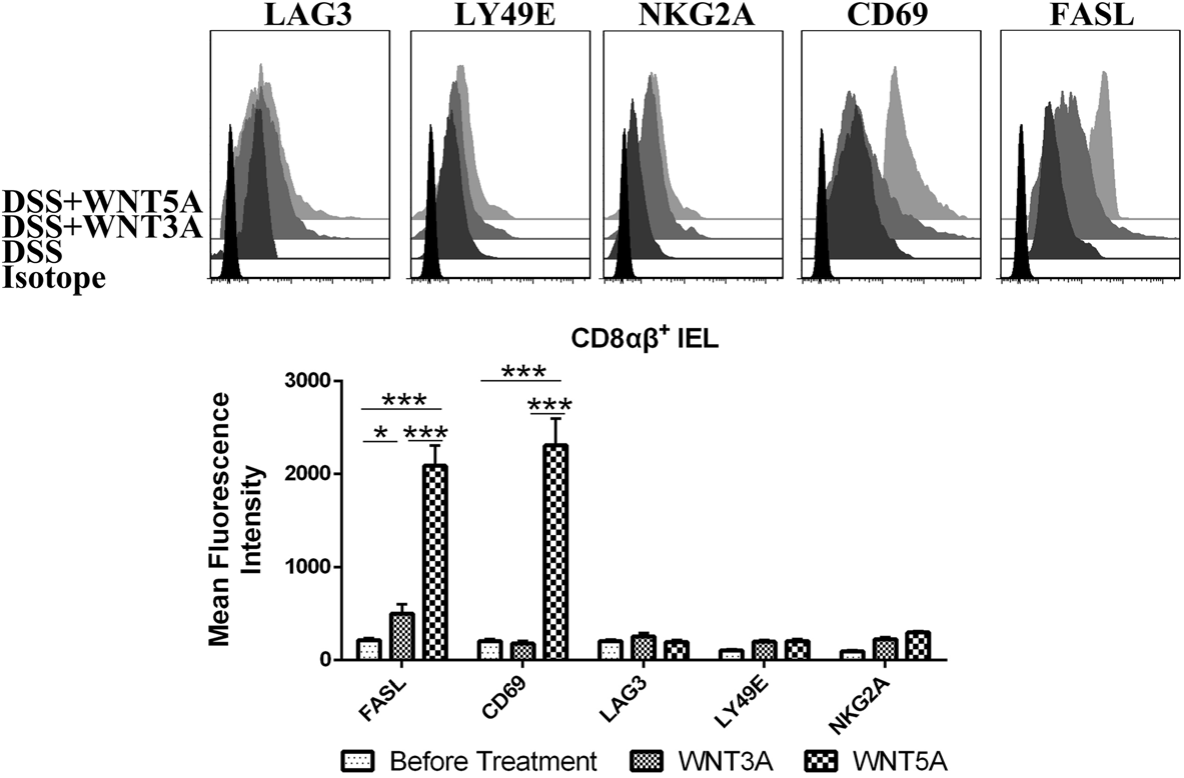
Immuno-phenotype of CD8αβ^+^ cells conditioned with WNTs. Sorted intestinal CD8αβ^+^ IELs from DSS colitis mice were treated with 0.4 ng/ml WNT3A or 0.4 μg/ml WNT5A for 24 h as indicated. Mean fluorescence intensity (MFI) was expressed as means ± SEM. Data represents three mice from two individual experiments. *p < 0.05, **p < 0.01, ***p < 0.005

To serve as additional controls we also investigated the response of CD8αβ + IELs after conditioned with WNTs. The antigen-experienced cytotoxic T cells protect the hosts against various infectious agents. WNT5A can significantly enhance the activation marker FASL and CD69. Canonical WNT signal modestly activated these cells to a lesser degree than the WNT5A, suggesting WNT pathways acting in a cell-type specific manner even within the IELs population (Fig. 3).

### Canonical and non-canonical WNT stimulants act in opposing manners

**Fig. 4:**
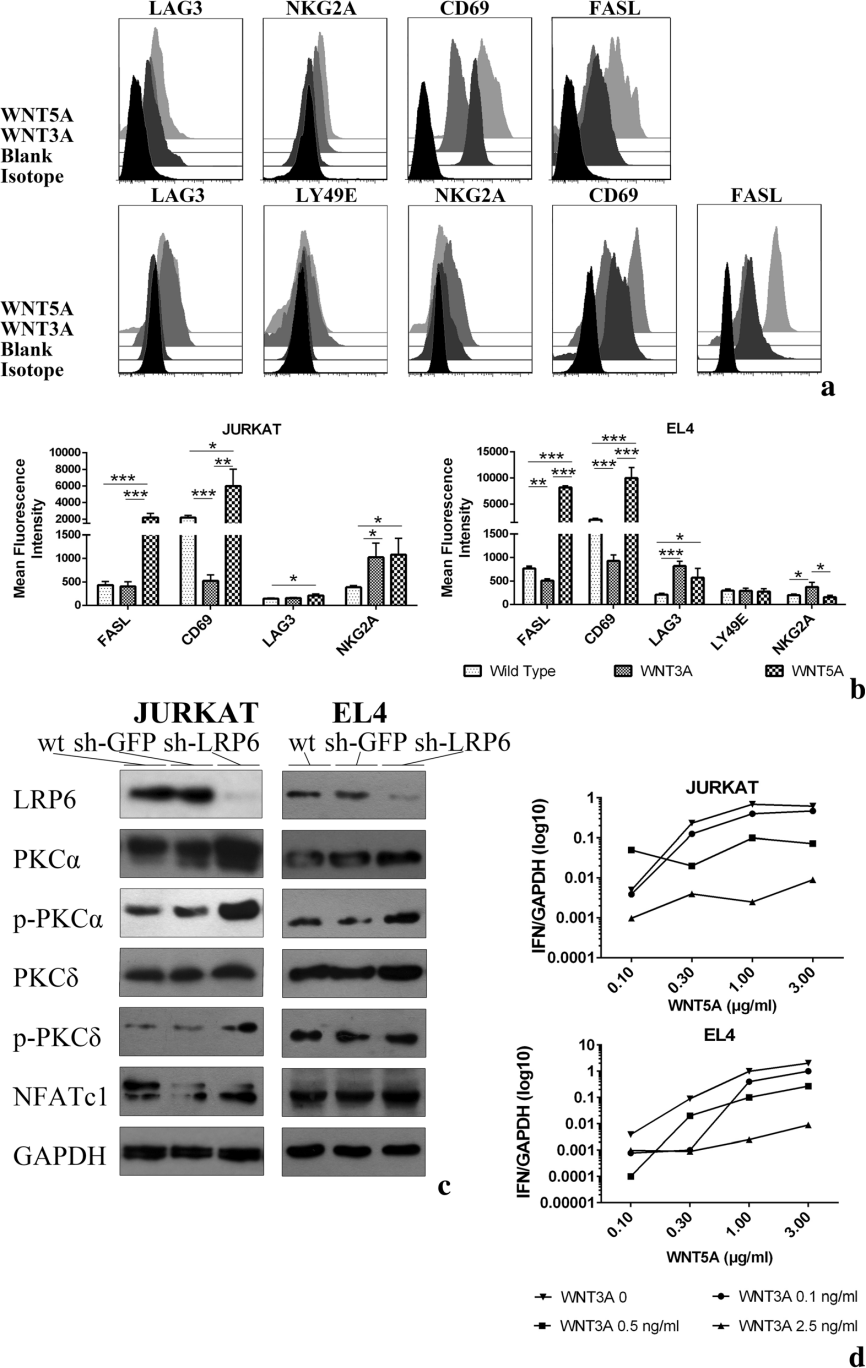
Immuno-phenotype of JURKAT and EL4 cells conditioned with WNTs. **a** Expression of surface markers in JURKAT (up) and EL4 (down) cells. Data represent three mice from two individual experiments. **b** Mean fluorescence intensity (MFI) was expressed as means ± SEM. Data represent from two individual experiments. **c** Western blot analysis demonstrates the greater activities of the WNT5A-PKC pathway in JURKAT and EL4 cells compared to wild type. **d** RT-PCR analysis of IFN of JURKAT (up) and EL4 (down). Cells were treated with different WNTs combination as indicated. mRNA expression was normalized to GAPDH expression. *p < 0.05, **P < 0.01, ***p < 0.005

We then examined the WNT signals’ effects on two T lymphocyte cell lines: the human originated JURKAT and C57BL originated EL4. WNT5A can significantly enhance the activation marker FASL and CD69 in both cell lines while only modestly affect the regulating markers. That is probably due to the devoid of tolerogenic ability in these two cell lines. On the contrary, canonical WNT signals continually exert an immune-modulating role when it dampens CD69 and promote LY49E expression in JURKAT cells. WNT3A can also decrease FASL and CD69 expression while increasing LAG3 expression in EL4 cells thus model them into an less immune-active status (Fig. 4a and b).

LRP-6 is a transmembrane co-receptor for Wnts that binds to Frizzled (FZD) receptors and thereby to the WNT/β-catenin signaling cascade whose normal activity have recently been shown to be critical for inhibition of non-canonical WNT pathway, including Rac1, PKC, RhoA and ROCK [18]. To establish the link between canonical WNT and non-canonical WNT pathway, LRP6 expression was knocked down in both JURKAT and EL4 cells using siRNA technique. Cells were then treated with WNT5A and WNT3A. As expected, LRP6 knockdown led to increased expression of non-canonical WNT proteins phospho-protein kinase C (PKC) α, PKCδ and their phosphorylated forms. These findings also associated loss of function of LRP6 with increased activation of nuclear factor of activated T cells (NFATc1), the non-canonical WNT effector which had been implicated in both Crohn’s Disease and Ulcerative Colitis [19] (Fig. 4c). WNT5A can trigger intracellular calcium flux, which can lead to the activation of PKC, calcium-dependent molecules such as calmodulin-dependent protein kinase II or calcineurin, and then NFATc1. Nucleo-translocation/activation of NFATc1 in lamina propria mononuclear cells was found in ulcerative colitis. As expected, with deactivated LRP6 and muffled WNT canonical pathway, both NFAT and its dephosphorylated form were boosted, implicating a greater ability to activate its downstream genes including IFN.

We next examined whether WNT3A can act against WNT5A in IFN production. Stimulation of both JURKAT and EL4 cells with recombinant WNT3A (0.1, 0.5, 2.5 ng/ml) showed a clear dose dependent inhibition of IFN production comparing to WNT5A alone (Fig. 4d),which was in compliance with NFATc1 and its dephosphorylation form protein level. These results implicate WNT3A acts in antagonist with WNT5A in activating PKCα-Cn-NFAT-IFN axis. WNT5A may skew T cells into a pro-inflammation characteristic while WNT3A can help maintain an immune-modulation phenotype.

### Interrupt WNT5A-PKC-NFATc1 pathway could curtail its immune-activating effect

**Fig. 5:**
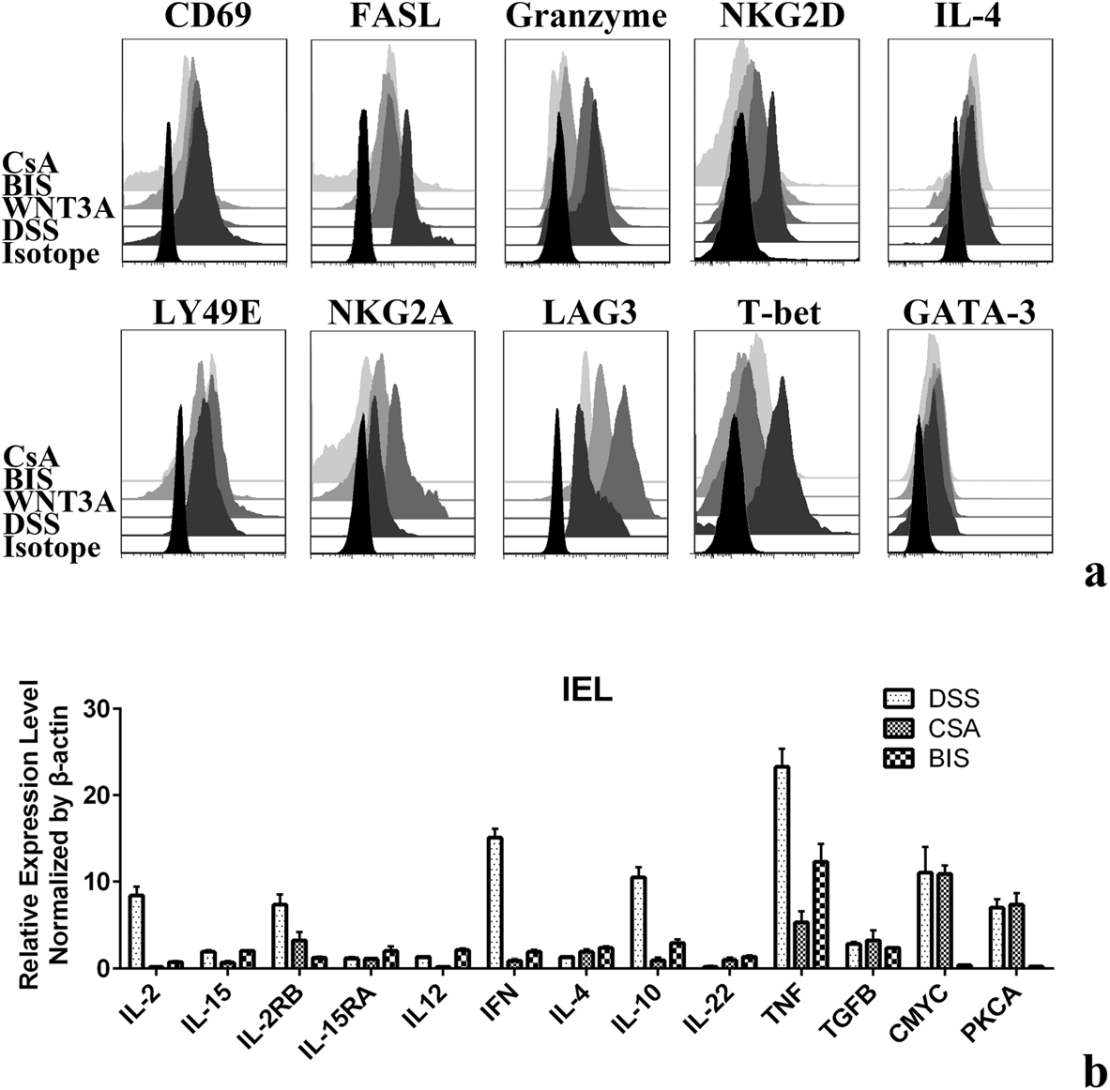
Markers and cytokines expression by CD8αα^+^ treated by WNT5A-PKC pathway inhibition. **a** Expression of surface or intracellular markers in CD8αα^+^ cells. **b** RT-PCR analysis of cytokines. Samples were normalized to β-actin expression. Data presented with means ± SEM from three independent experiments

Selective inhibitors were applied to further validate the role of PKCα-Cn-NFAT-IFN axis in DSS induced activated CD8αα^+^ IELs. 24 h of incubation with the potent and selective PKC inhibitor Bisindolylmaleimide (BIS) abated proinflammatory markers FASL, granzyme and NKG2D but not CD69 and IL-4. At the same time, using NFATc1 inhibitor CsA can significantly deactivate IELs as assessed by suppressed CD69, FASL, granzyme and NKG2D expression. However, comparing to WNT3A, both of the two inhibitors could only moderately endow an immune-regulatory trait as judged from LY49E, NKG2A and LAG3 expression. Since both granzyme and NKG2D are Th1 related immune molecules, we then looked in to the Th1 transcription factor T-bet expression after PKC and NFATc1 inhibition. In compliance with cell surface markers panel alterations above, it decreased after both PKC and NFATc1 inhibition. The Th2 cytokine IL-4 and transcription factor GATA-2 expression level were unaffected by the treatment (Fig. 5a).

A series of cytokines’ expression levels were also examined using rtPCR after PKC or NFATc1 inhibition. The downstream effectors C-MYC and PKCA were noticeably decreased after BIS employment together with inflammatory IL-2 and IFN as well as IL-2 receptor. TNF level were also slightly confined. CsA demonstrated an even greater ability to suppress immune response as measured from IL-2, IL-15, IL-12, IFN and TNF mRNA transcription level. Interestingly, inhibition of PKCα-Cn-NFAT axis alone could not boost IL-10 or TGFβ production. The above findings implicate activation of non-canonical Wnt signal in colon epithelial inflammation and identify the PKCα-Cn-NFAT-IFN axis as a potential target for drug development against inflammation (Fig. 5b).

## Discussion

Previous studies of β-catenin–dependent mechanisms in immune tolerance provided the rationale for assessing the role of WNTs in IELs function. In this study, we chose to examine WNT3A as a representative ligand that characteristically activated the canonical β-catenin pathway and WNT5A, which, in most cellular system, mediates non-canonical PKC signaling. In a lot of previous work, those two systems have been found to act opposing roles in stimulating immune responses. In this study, we were able to show that the canonical WNT signal and non-canonical WNT signal differentially induced anti- or pro-inflammatory immune responses in IELs. Selectively inhibiting the WNT5A-PKCα-NFAT axis could ameliorate the inflammation related processes within IELs. At the same time, siRNA knockdown of canonical WNT signaling co-receptor removed the brake and resulted in further activation of non-canonical WNT pathway. We used several most commonly assessed markers to conduct immune phenotyping in CD8αα^+^ IELs. LAG3 negatively regulates T-cell function and homeostasis [20, 21], and it is an essential component for the suppressive function of CD4^+^CD25^+^ regulatory T cells [22]. The LY49E NK receptor is a unique inhibitory receptor, presenting with a high degree of conservation among mouse strains and expression on both NK cells and colonic intraepithelial-localized T cells including CD8αα^+^ IELs. LY49E does not or only weakly recognize MHC-I, Sequestration of LY49E resulted in a 2-fold increase of the response of IELs to TCR stimulation [23, 24]. NKG2A is an inhibitory C-type lectin most commonly found on NK cells and some cytotoxic CD8^+^ T cells. The receptor–ligand binding of NKG2A to the MHC class Ib antigen HLA-E (human leukocyte antigen E; Qa-1b in mice) suppresses effector cell cytotoxic activity. NKG2A may also serve to prevent or reduce an inflammatory neutrophil-driven positive-feedback loop, via inhibition of cytokines (such as IL-6 and IL-17), thus subsequently impacting on overall neutrophil recruitment. In addition, NKG2A expression on NK cells protected mice from DSS-induced colitis development. Additional data from human studies supported a possible role of NK receptor expression in ulcerative colitis development and progression [25]. Although self-reactive CD8αα^+^TCRαβ^+^ natural IELs are normally immunologically quiescent, they have a potent antigen-experienced cytotoxic effector phenotype, which is characterized by high levels of granzymes, CD95 ligand (CD95L; also known as FASL) and CD69. Like activated splenic CD8^+^ cells, granzymes are secreted by IELs and induce apoptosis in target cells while FASL induces apoptosis following engagement of Fas on target cells [26].

Under normal conditions, CD8αα^+^ IELs are not self destructive, and evidence indicates that self-tolerance and regulatory features are associated with the molecules they secrete. TGF-β and IL-10 are critical participants in the maintenance of tolerance and immune homeostasis, acting on innate immune and stromal cells to suppress production of pro-inflammatory cytokines or chemokines and modulate immune responses of epithelial cell components nearby [27]. They also have important functions in protection at mucosal surfaces: IL-10 acts on epithelial cells to promote epithelial barrier integrity [28], and TGF-β stimulates IgA production [29] and acts on T cells to support the generation and survival of peripheral Foxp3^+^ Tregs [30]. Whether the TGF-β is secreted in biologically active form in this setting remains to be determined.

Newly found results show that the signature Th1 transcription factor T-bet can direct both the development of CD8αα^+^ IELs and its further differentiation and expansion in response to IL-15 [31]. In our study, the WNT5A signal seems to induce a Th1 immune response in IELs as characterized by a remarkable IFN production, up-regulation of granzymes and FASL [32]. We managed to find that the intracellular T-bet level also increased after non-canonical WNT activation while the Th2 master transcription factor GATA-3 was unchanged, indicating the significance of Th1-like pathway in their mature state functioning. However, we also observed an enhanced expression of intracellular NFATc1 and its dephosphorylated form, which was the hint of Th2 type of response. Yet we did not find the IL-4 production augmentation, which shall be result of NFATc1 transcriptional activation [33]. Still some other study showed that activation of the canonical WNT–Frizzle signal would induce the accumulation of β-catenin in Th2 cells and promoted GATA3 transcription [34], which was in contrast with our findings. Those diverse modifications suggest there may be a cell specific reaction to the WNT cascading thus further investigations are needed to address the exact intracellular signaling network reprogrammed by WNT ligands.

Recent studies indicate that besides basic duty of cell-fate specification, progenitor-cell proliferation and the control of asymmetric cell division, WNT proteins also regulate effector T-cell development, regulatory T-cell activation and dendritic-cell maturation. Growing evidence points to the WNT signaling pathway as a pivotal piece in the immune balance ranging from anti-tumor immunity to auto-immunity related disease [8, 15, 35]. WNT/β-catenin pathways in human melanoma may be involved in immune-suppression and immune-resistance in both induction and effector phases of antitumor immune responses. β-catenin-accumulated melanoma have activities to impair DC maturation and to induce possible regulatory DCs both in culture and in implanted mice model. Those β-catenin-overexpressed melanoma cells can also inhibit IFN-γ production by melanoma-specific CTLs in an IL-10-independent manner and is more resistant to CTL lysis in vitro and in vivo [36]. Other evidences showed that WNT ligand-mediated activation of β-catenin signaling in DCs was critical for promoting tolerance and limiting neuro-inflammation. Loss of LRP5/6-β-catenin-mediated signaling in DCs led to increased production of pro-inflammatory cytokines and decreased production of anti-inflammatory cytokines such as IL-10 and IL-27 [37]. Co-stimulation with LPS and WNT3A can lead to a significant increased IL-10 expression and a down-regulation of pro-inflammatory cytokines TNFα, IL-1B, and IL-6 in glial cells [38]. WNT/β-catenin signaling can also trigger anti-inflammatory effect in fibroblasts by selectively inhibiting the expression of a pro-inflammatory subset of IL-1β-induced NF-κB target genes [15].

However, the effects brought about by WNT3A has highly cellular specificity. The activation of WNT signaling reduced Treg-mediated suppression has been reported both in vitro and in vivo [39], possibly through inducing of expression of RORγt, the signature transcription factor of Th17 cells [40]. Those variances further emphasize the complex of immune cell fate determination.

On the other hand, several changes observed in WNT5A-treated cells have been associated with pro-inflammatory responses whereas WNT5A activates non-canonical signaling and can even antagonize the canonical pathway. For example, non-canonical WNT expression is increased during infection and inflammation: WNT5A is upregulated in arthritis synovial tissue, atherosclerotic plaques, psoriatic or wounded skin, and in inflammatory bowel disease [41]. In fact, the established role of non-canonical WNT-PKCα axis has prompt development of PKC inhibition in clinical setting. A well designed study proved that inhibition of PKCα pathway using Sorastaurin can stabilize Treg phenotype as evidenced by maintenance of high Foxp3 and CD25 expression, and lack of IL-17A and IFNγ production and has shown efficacy in clinical trials of psoriasis [42]. At the same time, restraining PKC pathway also demonstrated protection against ischemia-reperfusion injury and confer renal graft protection in rat models [43]. The general potent competence of Sotraustaurin to deactivate T cells has made it into Phase II renal and liver transplantation clinical trials.

Finally, although tolerance toward innocuous antigens from the diet or from resident non-invasive commensals is crucial for IELs to maintain the homeostasis of intestine, suppression of the immune response is undesirable in the context of tumor immunity. WNTs are frequently overexpressed in cancers [7], and their immune suppressive and tolerogenic effects on IELs could contribute to tumor genesis especially in the case of ulcerative colitis which has an estimated higher risk of developing inflammation related colorectal cancer [44].

## Conclusion

In this work we managed to demonstrate that canonical and non-canonical WNT signals act in opposing manners when determine CD8αα^+^ IELs immune status, as WNT5A can program those cells with a pro-inflammatory feature. Targeting non-canonical WNT pathway may be promising in tackling inflammatory bowel disease.

## Abbreviations

IELs: intraepithelial lymphocytes
WNT: Wingless-Int
UC: ulcerative colitis
NF-κB: nuclear factor κB
MFI: mean fluorescence intensity
FZD: frizzled
PKC: protein kinase C
NFATc1: nuclear factor of activated T cells, cytoplasmic 1
IFN: interferon
BIS: bisindolylmaleimide
TNF: tumor necrosis factor
